# Review of "Data Analysis with Open Source Tools" by Philipp K Janert

**DOI:** 10.1186/1758-2946-3-10

**Published:** 2011-03-24

**Authors:** Noel M O'Boyle

**Affiliations:** 1 Analytical and Biological Chemistry Research Facility, University College Cork, Western Road, Cork, Co. Cork, Ireland

## 

Cheminformatics has been defined as the application of informatics methods to solve chemical problems [1]. Such chemical problems are often represented in terms of data, be it activity data for a series of compounds or descriptor values for a compound library. While this new book from the O'Reilly stable is not aimed specifically at cheminformaticians, the subtitle of "A Hands-On Guide for Programmers and Data Scientists" makes it clear that the target audience includes any scientists whose day-to-day work involves analysing and interpreting data.

The book is broadly divided into four parts on Graphics: Looking at Data, Analytics: Modeling Data, Computation: Mining Data and Applications: Using Data. First of all, it should be noted that this is not a book about statistics (as Chapter 1 states explicitly). Neither is it a manual for numpy, Sage, matplotlib, Gnuplot, R and so forth, as might be implied by the title. Instead, Janert focuses on discussing data analysis methods and techniques in depth, rather than skimming topics by following a cookbook or tutorial approach linked to particular software. This is as it should be - there are already documentation and manuals available for all of these programs, and the reader is simply alerted to the availability of the software, its capabilities are described and some examples of use shown.

This is a real practitioner's book. Janert, a former physicist and software engineer, is a consultant in data analysis and mathematical modelling. He has taken his hard-won knowledge and tried to get it all down on paper for the reader's benefit. For example, in a chapter with the provocative title of "What you really need to know about classical statistics" he explains why introductory statistics textbooks seem to cover methods and topics at odds with the problems data analysts deal with day-to-day; essentially classical methods were developed at a time of small and expensive datasets and no computational power, and hypothesis testing focused on determining whether an effect existed. Today we have ample computing power and may be dealing with very large datasets; also, we are usually more interested in the size of an effect (practical significance) rather than just whether it exists (statistical significance).

Topics that could not be squeezed into a chapter proper have been placed in shorter "Intermezzos" at the end of each section. For example, a short section on "What about map/reduce?" at the end of "Mining Data" reminds the reader that the map/reduce methodology (much hyped recently) is not a clever algorithm to speed things up, but rather a piece of infrastructure that makes it convenient to implement algorithms that are trivially parallelisable.

On the negative side, any cheminformatician who has been involved with QSAR studies will already be familiar with the multivariate analysis methods discussed here (Chapters 13 and 14), although I liked the observation that "you will actually spend *more *time on data sets that are totally worthless" in relation to clustering algorithms. Also there are two chapters (out of 19) which will be of little interest as they focus on business intelligence and financial calculations, although even there the reader will find an introduction to the use of Berkeley DB and SQLite from Python, tools which I highly recommend. There are also cases where the author perhaps gives too much detail, but this is hardly a criticism - in a book of some 500 pages there is plenty of room.

Overall though, I heartily recommend this book to anyone working in cheminformatics whether they develop methods or apply them. Too often we rely on summary statistics such as mean and standard deviation and forget to actually look at the data. Graphical analysis gives you a feel for the data, and can often highlight problems, interesting features, or mistaken assumptions. After reading this book, you should be very aware of both the advantages and pitfalls of a wide variety of analysis methods but you will also be reminded that the goal of data analysis is not a picture or a number but insight.

## Competing interests

The author declares that they have no competing interests.
